# Towards human exploration of space: The THESEUS review series on nutrition and metabolism research priorities

**DOI:** 10.1038/npjmgrav.2016.29

**Published:** 2016-08-18

**Authors:** Audrey Bergouignan, T Peter Stein, Caroline Habold, Veronique Coxam, Donal O’ Gorman, Stéphane Blanc

**Affiliations:** 1 Anschutz Health and Wellness Center, Division of Endocrinology, University of Colorado, Anschutz Medical Campus, Aurora, CO, USA; 2 Université de Strasbourg, IPHC, Strasbourg, France; 3 CNRS, UMR7178, Strasbourg, France; 4 Department of Surgery, Rowan University, Stratford, NJ, USA; 5 Centre de Recherche en Nutrition Humaine d’Auvergne, Clermont-Ferrand, France; 6 Department of Health & Human Performance, Dublin City University, Dublin, Republic of Ireland

## Abstract

Nutrition has multiple roles during space flight from providing sufficient nutrients to meet the metabolic needs of the body and to maintain good health, to the beneficial psychosocial aspects related to the meals. Nutrition is central to the functioning of the body; poor nutrition compromises all the physiological systems. Nutrition is therefore likely to have a key role in counteracting the negative effects of space flight (e.g., radiation, immune deficits, oxidative stress, and bone and muscle loss). As missions increase in duration, any dietary/nutritional deficiencies will become progressively more detrimental. Moreover, it has been recognized that the human diet contains, in addition to essential macronutrients, a complex array of naturally occurring bioactive micronutrients that may confer significant long-term health benefits. It is therefore critical that astronauts be adequately nourished during missions. Problems of nutritional origin are often treatable by simply providing the appropriate nutrients and adequate recommendations. This review highlights six key issues that have been identified as space research priorities in nutrition field: in-flight energy balance; altered feeding behavior; development of metabolic stress; micronutrient deficiency; alteration of gut microflora; and altered fluid and electrolytes balance. For each of these topics, relevance for space exploration, knowledge gaps and proposed investigations are described. Finally, the nutritional questions related to bioastronautics research are very relevant to multiple ground-based-related health issues. The potential spin-offs are both interesting scientifically and potentially of great clinical importance.

## Introduction

Nutrition has several key roles during space flights from the basic nutritive intake to meet the metabolic needs of the body and to maintain the organism in good health, to the beneficial psychosocial aspects related to the meals.^1^ Because nutrition is the source of the energy, the precursors for synthesizing the functional body units (cells and their core constituents, the macromolecules proteins, DNA, RNA, cells, and so on), and provides a complex array of various co-factors such as micronutrients, including minerals and vitamins, to support enzymes activity and to optimize detoxification/repairment mechanisms, nutrition is central to the functioning of the body. Conversely, poor nutrition compromises most of the physiological systems.

Nutrition is likely to have a key role in counteracting many of the negative effects of space flight (e.g., radiation, immune deficits, oxidative stress, and bone and muscle loss). Nutritional deficiencies are often encountered with space flight. As missions increase in duration, any dietary imbalance will become progressively more detrimental. It is therefore critical that astronauts be adequately nourished during missions. Problems of nutritional origin are often treatable by simply providing the appropriate nutrients and recommendations.

Of particular concern for long-duration missions is the inability to maintain energy balance.^2–5^ Energy balance is the result of energy intake and expenditure. When energy intake exceeds energy expenditure, energy balance is positive resulting in creation of reserves of fat and eventually obesity. In contrast, when daily energy expenditure is greater than dietary intake, energy balance is negative leading to a depletion of fat and muscle stores and loss of body mass and eventually starvation. Energy requirements during space flight are similar to those on the ground.^2,6^ Yet, astronauts often consume less food than needed to cover their energy expenditure,^2–4^ which induces body mass loss. Although such energy deficits are physiologically tolerable for short-term missions because of the availability of body fat stores, a chronic negative energy balance becomes a significant detrimental issue jeopardizing health and performance for long-term missions.^3^ The causes of the energy imbalance are, however, not understood. A pre-requisite to advising astronauts on how to maintain energy balance is to know what the exact energy requirements are. It is also important to include the additional energy costs of any exercise countermeasures used when determining astronaut energy needs.

Food should actually provide a balanced diet and not empty calories. This is why energy, macronutrients, micronutrients, and vitamins requirements need to be evaluated for long-term missions, and nutrition countermeasures need to be tested. Bed rest is an appropriate ground-based model for most systems. For a Mars mission, there are technological challenges of providing a variety of palatable and nutritious foods, and ideally, some fresh foods. This is important not only for the obvious nutritive role of maintaining crew health, but also for the psychological aspects. Nutritional shortcomings can affect mood and behavior and psychosocial cohesion between the crewmembers.

Finally, the nutritional questions related to bioastronautics research are very relevant to multiple ground-based-related health issues, including chronic-age-related conditions. The potential spin-offs are both interesting from a technical point of view and clinically of great importance.

The project THESEUS funded in 2012 by the European Union aimed to develop an integrated life sciences research life roadmap enabling European human space exploration in synergy with the ESA strategy, taking advantage of the expertise available in Europe and identifying the potential of non-space applications and dual research and development. Fourteen disciplinary Expert Groups (EG) composed of key European and International experts in their field met four times and based their work on brainstorming sessions dedicated to identifying key issues in their specific field of knowledge. Key issues were defined as disciplinary topics representing challenges for human space exploration, requiring future attention in the future. These key issues were addressed to the scientific community through an online consultation; comments and inputs received were used to refine them and to consider knowledge gaps and research needs associated with them. Within the nutrition expert group of the THESEUS European project, six key issues related to exploration have been identified as space research priorities in the nutrition field. Each of these topics is briefly introduced followed by relevance for space exploration, knowledge gaps and proposed investigations. The earth benefits and transdisciplinary aspects of the nutrition field research in the context of space science are then described.

## The in-flight negative energy balance

### Relevance for space exploration missions

**Figure 1:**
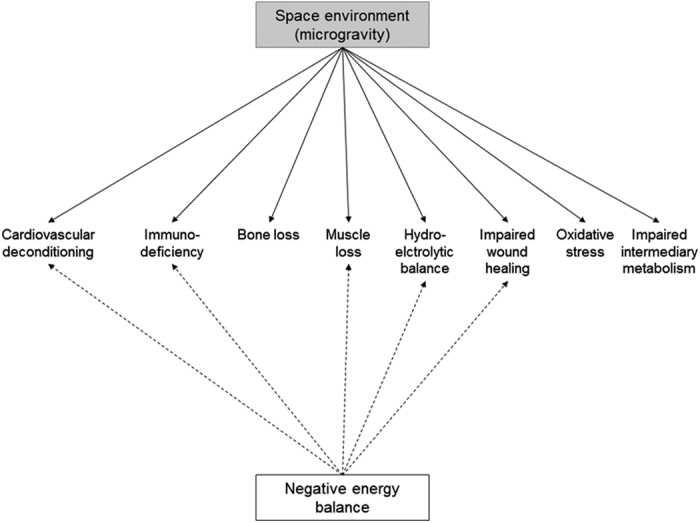
Deleterious adaptations induced by microgravity environment and energy deficit.

As stated in the introduction, a major medical issue of space flight is body mass loss of astronauts during the space missions. Among the four published studies that examined energy balance during space flights,^5,7–9^ three of them demonstrated a negative energy balance. Following a space flight of 16 days, Stein *et al.*^9^ noted in four astronauts an average energy deficit of 5.7±0.3 MJ per day, which can lead to a body mass loss up to 5 kg per month. Such an energy deficit is tolerable for short-term missions because of the high energy density of fat stores, but will have serious deleterious consequences over the long term if the energy imbalance is allowed to persist. A chronic energy deficit can jeopardize the health of the crewmembers and the success of missions. On the ground, a chronic negative energy balance induces impaired physical performances of muscle and cardiovascular functions and increased muscle fatigability, a greater susceptibility to infection, a compromised wound-healing capacity, an altered sleep and an overall reduced well-being. It has been suggested that a chronic energy deficit can exacerbate some of the deleterious physiological adaptations to space environment (Figure 1). For example, pilots who were following 2 months of partial fast had reduction in body weight (−2.7%) associated with diminution of maximal exercise capacity and orthostatic tolerance.^10^ This suggests energy deficit is implied in the cardiovascular deconditioning, characterized by these two physiological impairments, as it is observed in astronauts when returning to Earth after a space flight. However, in an elegant cross-over randomized study, Florian *et al.*^11,12^ did not observe an exacerbated negative effect of caloric restriction on top of 2 weeks of bed rest in adult men on orthostatic intolerance, central reflex activation, and metaboreflex. Of note, this study was conducted in nine participants only and the authors acknowledged it may have been underpowered. Stein *et al.*^9^ also showed that the decrease in protein synthesis is correlated to the energy deficit. Because muscle loss observed in astronauts is primarily due to a decrease in protein synthesis rather than to an increased protein catabolism, inadequate energy intake may contribute to muscle atrophy that is systematically observed during space flights. It has indeed been shown that chronic caloric restriction contributes to lower muscle^13^ and bone mass.^14–16^

Although the reasons for the inability of the astronauts to maintain a stable body mass requires further studies, the origin of the negative energy balance is likely twofold, i.e., too low energy intake and/or for too an high energy expenditure. On one hand, astronauts do not eat enough, they consume about 25–30% less calories during spaceflight than necessary to maintain body weight.^9,17^ On the other hand, exercise countermeasures performed to mitigate muscle and bone loss and cardiovascular deconditioning contributes to the negative energy balance. Physical exercise increases total energy expenditure (TEE) that needs to be balanced by greater energy intake that is not necessarily easy for the astronauts to consume. Exercise may further affect feeding behavior with an acute anorexia phenomenon, which would exaggerate the loss of appetite.

Given this chronic malnutrition, the needs for energy, and more specifically macronutrients and micronutrients, have to be evaluated with accuracy. Beyond this health aspect, the accurate estimation of in-flight energy needs can have important economic consequences. It has been estimated for a Mars mission of about 3 years and for a crew composed of 6 members that 22 tons of hydrated food, without taking into account water needs, should be carried in the space shuttle. Given that carrying 0.45 kg in space cost €10,000, even if water is totally recycled and food partially dehydrated, precise and accurate calculations of energy needs during space flight are obviously indispensable.

### Brief review of latest developments

It is extremely challenging to conduct an in-flight study to measure energy needs given the technical constraints and the other priority objectives of space missions. Furthermore, inhabited space flights are pretty rare and the number of astronauts rather restricted. The bed-rest ground analog model is therefore useful. To control energy balance of the astronauts, it is important to examine its different components, i.e., energy intake and expenditure. Daily TEE can be divided into three components: resting metabolic rate (RMR); the diet-induced thermogenesis and the physical activity energy expenditure. RMR corresponds to the minimal energy required to maintain the vital functions of the organism when at rest, fasted, and in thermoneutrality. It represents the main component of TEE. Diet-induced thermogenesis represents energy needed to digest, process, transport, and store nutrients following meal consumption. Physical activity energy expenditure is the amount of energy expended owing to any body movement. The calculation of daily energy needs take into account RMR and a coefficient proportional to physical activity of individuals. During bed-rest studies,^6^ energy needs of men and women were, respectively, evaluated to 1.47 and 1.45 RMR. These equations were similar to those derived in-flight from four astronauts by Stein *et al*.:^2^ 1.4×RMR. Consequently, energy needs seem to be equivalent to those on Earth and can be estimated on ground by using the bed-rest model. On average, energy needs in space are about 7.5 MJ per day and 8.5 MJ per day for a 55 kg woman and a 70 kg man, respectively.

Astronauts have in addition an intense physical exercise training aiming to counteract microgravity-induced muscle atrophy and bone demineralization. The energy expended as part of this exercise training needs to be taken into account for energy needs evaluation. In fact, likely it is the difficulty of estimating the energy cost related to exercise training that largely contributes to the in-flight energy deficit. One of the challenges is to know with precision how much the astronauts are exercising given that, like on Earth, the actual regime of exercise is usually much lower than the prescribed regime, both in terms of intensity and volume. As a result the actual energy regime onboard space stations is likely not as intense as prescribed. The exact exercise-related energy expenditure, however, needs to be objectively determined. In addition to these traditional components that are taken into account on Earth, astronauts perform extravehicular activity (EVA). A high number of EVA hours are planned for the missions to the ISS (~500 h). This type of physical activity has an estimated energy cost of 10.5 and 10.9 kJ/kg/h for women and men, respectively. When estimating energy needs, an additional 2.2 and 2.9 MJ per day therefore need to be added to the normal needs for 55 kg women and 70 kg men, respectively, assuming 6 h of EVA per day, which corresponds to the average daily time for EVA. As a result, energy needs would be 9.7 MJ per day for a woman and 11.4 MJ per day for a man. A space study is currently being conducted in the ISS to examine the contribution of each component of TEE and thus better understand the in-flight regulation of energy balance and estimate the daily energy requirements.

### Latest developments

A new generation of accelerometer-like devices have been developed for studies on Earth that are capable of defining the energy time budget of humans^18,19^ and wild animals.^20,21^ These devices coupled with other methods and instruments (i.e., heart rate monitoring, heat flow gauge, and so on) for measuring physical activity pattern and energy expenditure^22^ may give an accurate estimation of TEE in free-living conditions. The bioimpedance spectroscopy systems recently developed measure body composition^23^ and can therefore determine changes in body composition and thus energy balance changes over time.

### Knowledge gaps and research needs

The lack of an accurate method to assess in real-time the changes in energy balance precludes the monitoring of the nutritional status of the individual astronauts. Energy balance status needs to be assessed every other week and not less than once a month. The assessment should not, however, be confounded by changes in body composition (e.g., fluid shifts and electrolyte changes) or by physical activity patterns (duration and intensity) or in the rate of energy expenditure. The instruments used to measure these different outcomes should also not be disturbed by microgravity environment. Finally, the instruments should allow a non-invasive measurement of energy status and be cost-effective.

### Proposed investigations and recommendations

**Figure 2:**
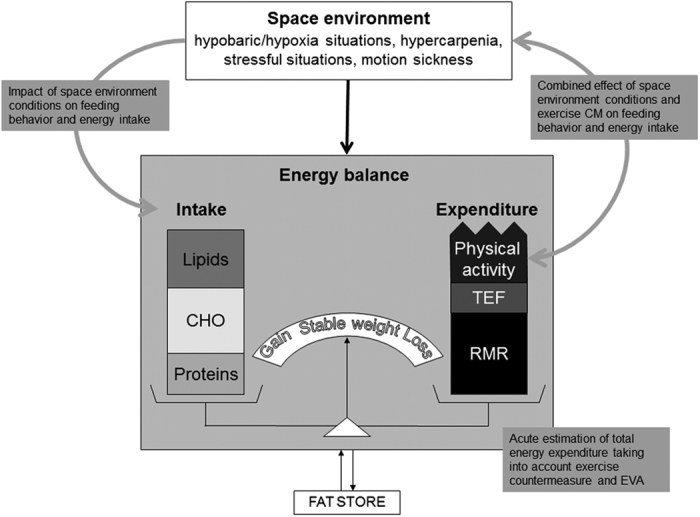
Energy balance regulation in space environment conditions. In gray are indicated the next questions to address to better understand how space environment conditions influence the two side of energy balance, i.e., energy intake and energy expenditure. CHO, carbohydrates; CM, countermeasure; EVA, extravehicular activity; RMR, resting metabolic rate; TEF, thermic effect of food.

Future investigations need to focus their efforts to detect early changes in energy status (Figure 2). This will require the validation of these methodologies, which include bioimpedance spectroscopy and latest generation of three-axial accelerometry and heart rate, against a gold standard method such as the doubly labeled water method. Such validation will have to be performed in flight to assess precision and accuracy of these methods in detecting in-flight changes in body composition and TEE (Figure 2). The validation of these instruments in flight is a crucial pre-requisite to planetary exploration. Regardless of the measure of the energy needs, we already know that astronauts should eat more to counteract the negative energy balance. The consumption of high-energy drinks classically used in sports nutrition should be tested as a countermeasure. There is some evidence that chrononutrition can modulate the anabolic capacity of the organism, a protein pulse-feeding pattern being more efficient than a protein spread-feeding pattern in whole-body protein retention (in elderly women).^24^ Using such a strategy to enhance body anabolism has not been considered as a possible countermeasure. We, however, should acknowledge that high protein intake has been associated with greater satiety and lower energy intake, which could contribute and exacerbate hypocaloric intake during spaceflight. This effect will have to be tested along with high-protein feeding.

If quantity has a role, the composition in macronutrients can also benefit or negatively have an impact on the body function. Overall, the in-flight nutritive needs are based on the daily recommended needs by the World Health organization (WHO) on Earth.^25^ A macronutrient composition of 15% proteins, 30% lipids, and 55% carbohydrates is therefore recommended on average. Some recent data, however, questioned this ratio and suggested that recommended protein intake was 41% underestimated.^26^ A greater protein intake may thus be more appropriate in space especially to counteract muscle. There is some evidence from bed-rest studies that supplementation with the essential branched amino acids can enhance protein synthesis and hence help counteracting muscle atrophy.^27,28^ In regard of the nature of fatty acids, like on Earth, unsaturated have to be favored over saturated fatty acids to prevent the development of metabolic alterations including the development of insulin resistance and inflammation.^29–31^

## Feeding behavior

### Relevance for space exploration missions

Part of the problem linked to the negative energy balance is a deregulation of eating behaviors. Astronauts eat 25–30% on average less than during pre-flight.^3^ The body mass loss of the astronauts observed over the first days of the spaceflight would be mainly owing to dizziness and motion sickness, phenomena experienced by 70% of the astronauts during their first space flight. The other causes are the decrease in food intake during the flights, an alteration of the anatomy of the GI tract and/or function and/or changes in appetite than may modify feeding behavior.

Eating behavior is a complex regulated process that represents a key issue on its own. To understand the factors that affect the feeding behavior in a space environment will help to manage the energy balance and will thus participate in the maintenance of the general health of the crewmembers.

### Brief review of latest developments

Much is now known about the regulation of food intake in humans and animals on Earth.^32,33^ Several good biomarkers for satiety and satiation, like leptin, GIP, GLP1, and ghrelin, are available and used in routine for investigations on feeding behavior.^34^ Those combined with imaging techniques, e.g., functional magnetic imaging resonance (fMRI) on Earth^35^ can provide critical information on how the above mentioned environmental factors, independently or combined, affect food intake regulation.

### Knowledge gaps and research needs

In the future, scientists need to answer a number of questions (Figure 2). We need to address the combined or independent effects of space environment elements (hypobaric/hypoxia situations, hypercarpenia, stressful situations, and motion sickness) on the regulation of food intake.

Exercise is known to have an anorexic effect and decrease appetite at least in an immediate manner.^36^ In a situation of intense exercise training, a decrease in appetite could occur.^36^ One of the related questions therefore is the impact of exercise countermeasure on feeding behavior. The respective effect of the different terms of the physical activity training, i.e., intensity, duration, volume, type of exercise, and so on, on appetite and hunger have to be taken in consideration. The lack of data on this topic is, however, not limited to space science and more investigations are also needed on Earth to address the direct relationship between exercise and food intake.

Furthermore, the interaction between exercises of different intensities, hypobaric/hypoxia situations, and hypercarpenic environment have to be tightly investigated on feeding behavior and appetite regulation of astronauts.

### Proposed investigations and recommendations

Future investigations need to address the impact of the different elements of the space environment, independently and combined, on feeding behavior. Numerous facilities exist that could allow to separate out the influence of the environmental factors observed in space on feeding behavior regulation. These include Concordia station, ISS itself and submarines. There is a dearth of in-flight and ground-based flight model data. As stated above, the main goal is to have astronauts eating more. The consumption of high-energy drinks classically used in sports medicine and the use of chrononutrition that has been shown to increase the anabolic capacity of the organism should be tested as a countermeasure. On the other side of the problem, an improvement in palatability of the items proposed by the space food industries need to be given more attention.

## Metabolic stress

### Relevance for space exploration missions

Metabolic stress, characterized by an increase in metabolic rate (hypermetabolism) leading to catabolism in association with protein use for providing energy and a suppressed immune system, is observed during space flight. Metabolic stress is known to affect the major body systems. It will certainly pose problems during exploration as a large body of data in clinical nutrition shows that it is a strong predictor of type 2 diabetes and cardiovascular diseases.^37–40^ In addition, the related oxidative stress and inflammation have been recently suggested to be involved in the progression of muscle disuse atrophy^41^ and bone loss.^42^

### Brief review of latest developments

As highlighted in some recent reviews,^37–40^ there is an extensive body of data available from recent clinical nutrition research. This concerns insulin resistance and lipotoxicity, type 2 diabetes, obesity, metabolic syndrome, cardiovascular risks, muscle function, and bone health. There, however, is much debate on the causes and subsequent development of metabolic stress. Sedentary behaviors and physical inactivity likely has a key role but further studies are required. In the context of human space missions, few data have been obtained in either actual or simulated weightlessness. During the Apollo space mission, astronauts developed insulin resistance in association with microvascular injuries and peripheral endothelial dysfunction. The potential underlying mechanisms that have been proposed based on observations made during space flights are magnesium deficits as indicated by excess C-peptide excretion and reduced cyclic-GMP (a second messenger of nitric oxide—NO),^43^ the development of vascular inflammation as indicated by reduction in both vascular endothelial growth factor and platelets,^44,45^ and systemic inflammation as indicated by increase in interleukin 6 excretion^46^ from the first day of spaceflight. Few recent bed-rest studies have investigated other potential mechanisms by using more sophisticated methods. The bed-rest-induced insulin resistance has been associated with reduced content and/or activity of key proteins involved in glucose transport in the myocyte and storage as glycogen,^47^ decline in insulin-stimulated glucose disposal,^47^ impairment of nonoxidative glucose metabolism,^48^ and also with reduced rate of lipolysis,^48^ and impaired microvascular function.^49^ So far, no increases in circulating inflammatory markers has been observed suggesting that systemic inflammation^49^ is likely not responsible for vascular dysfunction. These bed-rest studies were, however, of short term (from 5 to 10 days) and chronic effects of simulated or actual microgravity on the potential mechanisms underlying alterations of insulin sensitivity as well as changes in oxidative stress and inflammation still need to be studied.

### Knowledge gaps and research needs

In the context of spaceflight, an unresolved issue is clearly the functional consequences—both short- and long term of the development of insulin resistance. The interaction between insulin resistance, metabolic stress, inflammation, and oxidative stress has to be determined.

### Proposed investigations and recommendations

Fundamental research has to be conducted during bed-rest studies and during basic clinical science protocols to investigate whether/how metabolic stress can be alleviated with macronutrients and micronutrients supplementation. Further work is required here, especially into the underlying mechanisms.

## Micronutrients deficiency

### Relevance for space exploration missions

Prolonged micronutrients deficiency is clearly associated on the ground with numerous functional alterations.^50,51^ Malnutrition has also been observed in space.^52^ Inadequate intake of minerals (calcium, potassium, and sodium) and of oligo-elements (iron) in addition of deficiency in vitamins K and D have been observed.^52^ However, results obtained during space flights are contrasting. It was recently observed that plasma phylloquinone concentrations, urinary γ-carboxyglutamic acid, a measure of vitamin K-dependent protein turnover, and serum undercarboxylated osteocalcin, a measure of vitamin K function, were generally unchanged in response to flight,^53^ which do not support the need for vitamin K supplementation as a bone loss countermeasure during spaceflight. In the context of planetary exploration a clear evaluation of the micronutrient status during long-term space flight is therefore required to maintain health and more specifically to maintain muscle and bone mass, insulin sensitivity, to reduce oxidative stress, and lipotoxicity. It is also possible that micronutrients mitigate to some extent the deleterious consequences of radiation.

### Brief review of the latest developments

Many ground-based studies have well documented the importance of avoiding micronutrient deficiencies.^50^ Any potential problems can be avoided by advising the consumption of nutrient dense foods suppliers of micronutrients such as fruits or vegetable, or by taking a daily multi-vitamin/mineral supplements capsule.^54^ Such strategies could counteract the microgravity-induced physiological maladaptations. On Earth, supplementation in vitamin K has been shown to normalize the undercarboxykated levels of osteocalcin suggesting that supplementation in vitamin K may correct this abnormality and help preserving bone loss in astronauts. High calcium intake and vitamin D supplementation during space flights have surprisingly not conferred benefits on bone metabolism, but vitamin K supplementation has been shown to counteract the reduction in bone formation^54^ likely by re-establishing the undercarboxykated levels of osteocalcin.^55^ Other agents that have both anti-resorptive and anabolic effects on bone may be needed to stabilize calcium balance and bone metabolism.^56^ In a rat study, we recently tested whether a supplementation in resveratrol, a natural polyphenol known to act on numerous pathways involved on the deconditioning syndromes, could be used as a nutritional countermeasure to prevent muscle, metabolic, and bone adaptations.^57^ Unexpectedly, we observed that resveratrol treatment maintained a net protein balance, soleus muscle mass, and soleus muscle maximal force contraction, mitochondrial oxidative capacity, and prevented the development of oxidative stress and insulin resistance. Although resveratrol has strong potency in rodents, its effect on human metabolism and physiology has so far not been as unanimous.^58–60^ Several other micronutrients, like vitamin E^61^ or omega-3 fatty acids,^62^ were, however, reported to counteract to some extent some of the space adaptations.

### Knowledge gaps and research needs

So far, the state of general micronutrients (vitamins, minerals, and microconstituents) during space flight has been poorly investigated, especially during long-term missions. Results with regard to the effects of vitamin K supplementation and the changes in undercarboxylated osteocalcin need further investigations. In the area of nutritional supplementation of micronutrients, the primary open question is the applicability of population-based ground data. A major gap is whether target-specific micronutrients (e.g., resveratrol, creatine, omega-3 fatty acids, vitamin E, and so on) might prove to have unique benefits during spaceflight.

### Proposed investigations and recommendations

Long-duration bed-rest studies, and fundamental science, and of course studies during actual spaceflight to collect the data are the best direction to investigate this key issue. If needed, targeted micronutrient supplementation (e.g., resveratrol, creatine, omega-3 fatty acids, and so on) should be evaluated as countermeasures in tightly controlled experiments. The independent and combined effect of these different micronutrients should also be considered, as well as many biological targets. Metabolomic approaches would allow to search, without *a priori* hypothesis, the impact of such treatments on the entire body.

## Alterations of gut microflora

Perturbation in gut microflora is an understudied area on the ground that is increasingly recognized as important.^63,64^ Alterations in the gut microflora may affect nutrient absorption, have an impact on gut secreting peptides, and thus metabolism and food intake as well as immune status.^65–68^

### Relevance for space exploration missions

If the gut microflora is altered during space flight, this could potentially have an impact not only on energy intake and assimilation, and thus energy balance, but also on intermediary metabolism as well as immune system. Altogether, the maintenance of gut microflora can be important for the general health of the crewmembers.

### Brief review of latest developments

This is a very new research field, but the accumulated data do suggest major impact on health, particularly on intermediary metabolism, feeding behavior and immune status. Although there is some evidence that probiotic supplementations can benefit those with disease associated imbalances of the gut microbiota, a very recent review has shown no consistent effects of probiotics supplements containing live bacteria in healthy people.^69^

### Knowledge gaps and research needs

So far, the effect of microgravity on the gut microflora, bacterial diversity, and so on, has not been investigated. Because it is unclear whether astronauts develop imbalances in gut microflora or not, hypotheses about the potential effects of probiotic supplementation are difficult to propose. Therefore, there is critical need for the relevant data.

### Proposed investigations and recommendations

Bed-rest and fundamental science, and of course studies during actual spaceflight are the best direction to investigate this key issue. Prebiotics/probiotics supplementation might prove to be an efficient countermeasure.

## Fluid and electrolyte imbalance

The maintenance of fluid and electrolyte balance is required to maintain whole-body physiological homeostasis and thus to optimize performance.

### Relevance for space exploration missions

Alterations of the fluid and electrolyte balance in response to short-term exposure to microgravity have been observed in the past.^70^ Such alterations sustained over the long term would likely have a negative impact on the health of the crewmembers and thus jeopardize the success of the mission.

### Brief review of latest developments

No recent data are available, especially for long-term missions. However, a large body of data on body-fluid regulation is available from short-term space missions (e.g., EuroMIR94—30 days and MIR97—19 days). A thorough compilation of these results is out of the scope of the present review and can be found in other past reviews.^71–77^ In brief, in absence of gravity central blood volume expands owing to greater venous return and is accompanied by increased renal excretion rates of water and sodium under weightlessness conditions. Changes in excretion rates are under the control of neuroendocrine factors and cardiovascular reflexes. Surprisingly, astronauts, contrary to subjects submitted to head down tilt bed rest, display a fluid and sodium retaining state^73^ with activation of the sympathetic nervous system and changes in sodium/hydrogen exchange on glycosaminoglycans.^74^ The activation of these sodium-retaining humoral systems, such as renin–aldosterone and catecholamines, during space flights may contribute to a new steady-state of metabolic balances characterized by greater levels of body sodium than what is observed on Earth during simulated weightlessness. On the contrary, cumulative water balance and total body water content remain stable during space flight if hydration, energy intake, and muscle mass are maintained at an acceptable level.^73^

### Knowledge gaps and research needs

The long-term fluid and electrolyte adaptations to space flight are unknown. Specifically, the underlying mechanisms of this dissociation between water and sodium regulations in weightlessness are still poorly known. Salt intake may be an important factor influencing the renin–angiotensin–aldosterone system and thus fluid and electrolyte retention. The circadian rhythm of cortisol has also likely contributed to these changes, and little is known yet about the influence of spaceflight on circadian rhythms.

### Proposed investigations and recommendations

Spaceflight studies are the only option to investigate such adaptations. Hydration and mineral supplementations are the more potent countermeasure to test and apply. Further attention must be paid to salt intake, changes in circadian rythms during spaceflight, and its impact of body fluid and electrolytes.

## Trans-disciplinary aspects

As explained in the introduction, nutrition has an impact on multiple physiological systems and is therefore crucial for the optimal function of the organism. Investigations on the key issues highlighted in this review will thus provide relevant data for other fields of space research. On the ground, consequences associated with long-term energy imbalance include alterations in mood, physical and cognitive performance,^78,79^ stress regulation, bone and muscle mass,^3,78–80^ cardiovascular function, and immunology.^81–84^ Exercise countermeasure protocols should be established in relation to nutritional guidelines in order to reach a stable energy balance over the period of the space mission.^3,34,85^ When establishing guidelines on food intake, both in terms of quality and quantity, data from investigations on changes in gut microflora and its impact on energy balance regulation will also have to be kept in mind. Furthermore, the food industry and research on food supply will have to take into account the impact of food and meals on feeding behavior. Because food has a strong psychosocial role and can help to enhance the cohesion between the crewmembers, nutrition and feeding behavior will have to be taken in account for psychosocial status and well-being of the astronauts. There is a growing body of evidence suggesting oxidative stress and low-grade inflammation have a causal role in muscle disuse atrophy^41,86^ and bone loss.^42^ The development of insulin resistance seems to preclude the decrease of resistance to fatigue, which suggests that it may directly have an impact on performances. Micronutrients seems to have even a broader impact on physiology, as they are involved in intermediary metabolism, regulation of insulin sensitivity, the control of oxidative stress and lipotoxicity, and many signaling cellular pathways, which impact bone and muscle mass as well as muscle performances. Similarly, fluid and electrolyte imbalance could indirectly have an impact on musculoskeletal, kidney, vascular, and immune systems. Investigations on metabolic stress, fluid and electrolyte balance, and micronutrient needs will thus help gaining a better and more integrative understanding of human physiology in space, as well as on the ground.

**Figure 3:**
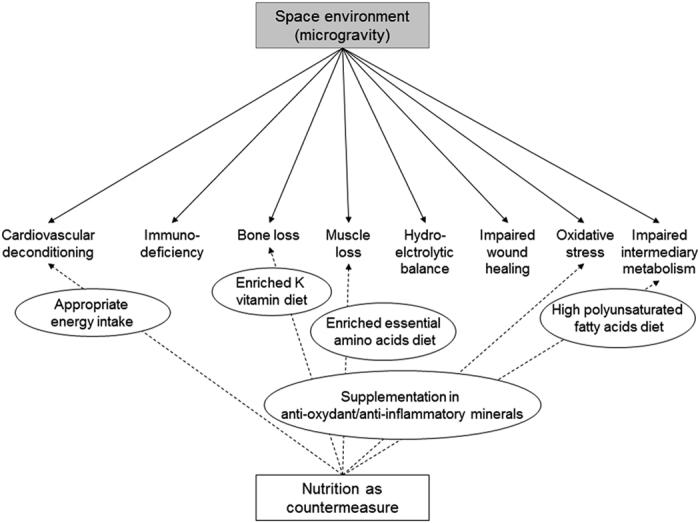
Nutrition countermeasure to mitigate deleterious metabolic adaptations induced by space environment.

Given the central role of nutrition in body function, malnutrition can have an impact on a number of physiological systems, and also when considered a countermeasure can benefit several body functions (Figure 3). As explained above, nutritional countermeasure such as supplementation in anti-inflammatory/anti-oxidant micronutrients, including vitamins E, K, D, polyphenols, polyunsaturated fatty acids and others, in addition to the more traditional protein/branched essential amino acids supplementation, is a very promising countermeasure that needs to be fully investigated. Keeping in mind that the good countermeasure is the countermeasure that maintain the integrity of physiologic systems without impairing one while favoring another one. For example, high protein intake likely exacerbates bone loss. Protein supplementation has indeed been shown to decrease blood pH through the oxidation of sulfur amino acids and thus aggravate bone loss.^87^ The amount of protein should therefore be attentively regarded when developing nutrition countermeasure.

## Earth benefits and applications

**Figure 4:**
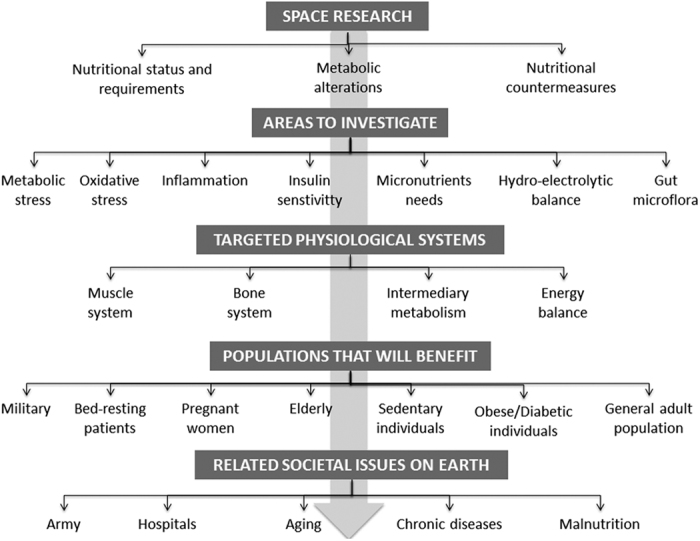
Summary of Earth benefits related to Space research.

The sophisticated research conducted in the field of space research has multiple Earth benefits (Figure 4). Being able to manage energy balance for an astronaut requires completing the development of an accurate system for nutritional status monitoring in space. This would be of tremendous interest to multiple groups on the ground, ranging from the military to the physicians working with obese patients and hospital doctors dealing with patients who are for one reason or another unable to eat normally, or with elderly people. Malnutrition among hospitalized patients remains a critical problem today. It contributes to increased morbidity, mortality, and increased time in the hospital and hence the costs of health care. Investigations on gut microflora will give some novel data about the effect of physical inactivity on the microflora and its impact on metabolism. Research on micronutrients and metabolic stress requirements will likely foster strategies to maintain bone and muscle mass, to control sensitivity to insulin effects, oxidative stress and lipotoxicity in patients, sedentary individuals and the elderly. Research on fluid and electrolyte balance will have strong benefits on Earth as similar alterations of fluid and electrolyte balance to that observed in microgravity conditions are observed during exercise and altitude challenges, and also during anorexia and adaptation to a high-protein diet in the sick patient. Given the general adoption of sedentary behaviors by generalized population in the westernized countries, as well as in the emergent countries, most of these studies will provide new data on the role of sedentariness in the development of chronic metabolic diseases that are reaching alarming prevalence in the world. In summary, studying nutrition in space will help promote health of the overall population on Earth.

## Conclusions

In the previous bioastronautic research, nutrition has never been a high priority, given the relative short duration of flights. With the construction of the International Space Station and the future long-term missions (e.g., Moon and Mars), the interest in nutrition as a countermeasure has markedly increased. Indeed, an increasing body of data show a direct relationship between nutrition and the deleterious adaptations to space with regard to both energy balance and regulation of the energy substrate (fuel mix) oxidation. Investigations on functional adaptations to actual or simulated microgravity need to include nutrition as an important variable in interpreting the observed results. One aspect that will also need more attention in the future is the influence of gender and the specific nutritive needs for men and women. Controlling for nutrition as a variable was often over-looked in the past, but will benefit health both in Space and on Earth in the future.
